# New Genomic Insights into “*Entotheonella*” Symbionts in *Theonella swinhoei*: Mixotrophy, Anaerobic Adaptation, Resilience, and Interaction

**DOI:** 10.3389/fmicb.2016.01333

**Published:** 2016-08-25

**Authors:** Fang Liu, Jinlong Li, Guofang Feng, Zhiyong Li

**Affiliations:** Marine Biotechnology Laboratory, Department of Bioengineering, State Key Laboratory of Microbial Metabolism–School of Life Sciences and Biotechnology, Shanghai Jiao Tong UniversityShanghai, China

**Keywords:** sponge symbiont, “*Entotheonella*”, metagenome binning, mixotroph, resilience, interaction

## Abstract

“*Entotheonella*” (phylum “Tectomicrobia”) is a filamentous symbiont that produces almost all known bioactive compounds derived from the Lithistida sponge *Theonella swinhoei*. In contrast to the comprehensive knowledge of its secondary metabolism, knowledge of its lifestyle, resilience, and interaction with the sponge host and other symbionts remains rudimentary. In this study, we obtained two “*Entotheonella*” genomes from *T. swinhoei* from the South China Sea through metagenome binning, and used a RASTtk pipeline to achieve better genome annotation. The high average nucleotide index values suggested they were the same phylotypes as the two “*Entotheonella*” phylotypes from *T. swinhoei* from the Japan Sea. Genomic features related to utilization of various carbon sources, peptidase secretion, CO_2_ fixation, sulfate reduction, anaerobic respiration, and denitrification indicated the mixotrophic nature of “*Entotheonella.*” The endospore-forming potential along with metal- and antibiotic resistance indicated “*Entotheonella*” was highly resilient to harsh conditions. The potential for endospore formation also explained the widespread distribution of “*Entotheonella*” to some extent. The discovery of Type II (general secretion pathway proteins and the Widespread Colonization Island) and Type VI secretion systems in “*Entotheonella*” suggested it could secrete extracellular hydrolases, form tight adhesion, act against phagocytes, and kill other prokaryotes. Overall, the newly discovered genomic features suggest “*Entotheonella*” is a highly competitive member of the symbiotic community of *T. swinhoei*.

## Introduction

The study of sponge microbiology has long been driven by the need for marine drug discovery (Piel et al., 2004; Wilson et al., 2014). Symbionts of marine sponges have become a fascinating field, due to their fidelity to hosts, unique phylogenetic patterns, importance in biogeochemical cycles, and great biotechnology potential (Taylor et al., 2007; Hentschel et al., 2012). To date, more than 50 phyla of microbial symbionts have been discovered from marine sponges based on pyrosequencing (Webster et al., 2010; Schmitt et al., 2012; Reveillaud et al., 2014). However, most sponge microbial symbionts remain uncultured, which limits our understanding of their metabolism and functions. Metagenome binning represents an approach that overcomes the difficulty of cultivating microbes and can reveal the unknown genomic features of uncultured microbes (Albertsen et al., 2013). Application of metagenome binning to sponge microbiology has yielded four draft genomes of “*Candidatus Synechococcus spongiarum*” (Gao et al., 2014; Burgsdorf et al., 2015), three draft genomes of autotrophic symbionts from deep-sea glass sponge (Tian et al., 2016), and two draft genomes of candidate genus “*Entotheonella*” (Wilson et al., 2014), which have greatly extended our knowledge of the functions of uncultured symbionts in sponges.

*Theonella swinhoei* and its Gram-negative, filamentous symbiont, “*Entotheonella*,” represent the most prolific and well-studied host–symbiont pair in terms of natural product isolation, chemical localization, symbiont identification, and PKS/NRPS gene cluster studies (Bewley et al., 1996; Bewley and Faulkner, 1998; Schmidt et al., 2000; Piel et al., 2004; Wilson et al., 2014; Ueoka et al., 2015; Freeman et al., 2016). A recent major advancement was the recovery of “*Entotheonella*” genomes from the metagenome of *T. swinhoei* from the Japan Sea, namely “*Candidatus Entotheonella factor*” TSY1 and “*Candidatus Entotheonella gemina*” TSY2 (Wilson et al., 2014). The genomic analysis provided confirmative evidence that almost all bioactive polyketides and non-ribosomal peptides derived from *T. swinhoei* are synthesized by “*Entotheonella.*”

Nonetheless, compared with the comprehensive knowledge of the secondary metabolism repertoire of “*Entotheonella*,” little is known about its other metabolic features. The large genome size (>9 Mb) and candidate taxonomic status make its genome annotation very difficult. Additionally, the wide distribution of “*Entotheonella*” in sponges raises more questions about its genetic diversity and ecological roles (Wilson et al., 2014). Clearly, more “*Entotheonella*” genomes are needed for a better understanding of this uncultured sponge symbiont.

In this study, two “*Entotheonella*” genomes were obtained from the metagenome of the sponge *T. swinhoei* from the South China Sea, which showed high coherence to the two other genomes derived from the Japan Sea (Wilson et al., 2014). Based on the annotations of the four genomes, the lifestyle of “*Entotheonella*,” resilience, and interaction with the sponge host/other microbes are discussed. The results suggest that “*Entotheonella*” is a highly competitive member of the symbiotic community of *T. swinhoei*.

## Materials and Methods

### Sample Collection, DNA Extraction, and Metagenome Sequencing

Individuals (*n* = 3) of *T. swinhoei* (yellow interior) were randomly sampled by scuba diving within a 15 m radius at approximately 10 m depth near Yongxing Island (112° 20′ E, 16° 50′ N) in the South China Sea, and were morphologically identified by Prof. Jinhe Li at the Institute of Oceanology, Chinese Academy of Sciences. Sponge tissues were quickly rinsed with sterile artificial seawater then cut into small pieces thinner than 5 mm. Subsequently, the specimens were fixed in RNA Later^®^ (Qiagen, Hilden, Germany) at 4°C for 8 h. The fixed specimens were then transported to the lab on ice. Total DNA was extracted with a QIAGEN DNeasy Tissue Mini kit (Qiagen), following the manufacturer’s instructions. The integrity of DNA samples was inspected by 0.8% agarose gel electrophoresis. DNA samples of good quality (concentration >50 ng/μl, 1.8 < *A*_260_/*A*_280_ < 2.0) were pooled and used for metagenome sequencing.

Metagenome sequencing was carried out at Genewiz, Inc (Beijing, China). In brief, a library with a 350 bp insert length was constructed and sequenced on a HiSeq 2000 (Illumina, USA), following the manufacturer’s instructions. Metagenome reads in FASTQ format (approximately 40 Gb) were trimmed using a minimum Phred score of 20 and a minimum length of 36, by Trimmomatic 0.32 (Bolger et al., 2014), allowing no ambiguous nucleotides or adaptors. Finally, about 28 Gb reads (125,866,412 paired-end reads and 47,841,730 single-end reads) were fed into metaVelvet for assembly (kmer = 57, minimum contig length = 300 bp; Namiki et al., 2012). The metagenome assembly used for metagenome binning included 371,342 contigs with a minimum length of 500 bp.

### Metagenome Binning, Taxonomic Assignment, and Genome Annotation

MetaBAT, an automatic metagenome binning software package, was used for metagenome binning (Kang et al., 2015). Metagenomic contigs no shorter than 2 kb were binned based on empirical probabilistic genome abundance and tetranucleotide frequency. According to the recommended protocol, the sensitive mode was applied first to recruit contigs then the specific mode was applied to strip off non-targeting contigs. As rRNA operons were often lost in the binning process due to their high coverage, a whole-genome tetra-correlation-search-based platform, JSpeciesWS Online, was used to find the targeting genomes (Richter et al., 2016). Average nucleotide index (ANI) was calculated to determine whether two genomes were closely related, using the script ani.rb with default settings^1^. The completeness of binned genomes was estimated based on the presence/absence of 106 essential bacterial genes, using the script HMM.essential.rb^1^.

The metagenome bins and publicly available “*Entotheonella*” genomes (AZHW01000000 and AZHX01000000) were annotated using a RASTtk pipeline based on the SEED subsystem (Brettin et al., 2015). Primary coding sequence (CDS) prediction and annotation was done by the scripts rast-call-features-CDS-glimmer3, rast-call-features-CDS-prodigal, and rast-annotate-proteins-kmer-v2, followed by rast-annotate-proteins-kmer-v1 –H to further annotate hypothetical proteins. Additionally, rast-call-features-ProtoCDS-kmer-v2 and rast-call-features-ProtoCDS-kmer-v1 were applied, as gene calling might have been missed in some regions. The PATRIC web service was used as the primary comparative analysis platform, in which KEGG pathways were implemented (Wattam et al., 2014). Bidirectional BLAST analysis was used to define the core gene set and singletons (genes without any hit against any other genome than their own) of four “*Entotheonella*” genomes based on the EDGAR platform (Blom et al., 2009). In bidirectional BLAST analysis, “*Candidatus Entotheonella factor*” TSY1 was used as reference owing to its high completeness. Scripts used for metagenome binning and genome annotation are provided in the Supplementary Information.

### Phylogenetic Analysis of Phosphoenolpyruvate Carboxylase (PEPC)

The BLASTP program was used to determine the sequences that were most closely related to “*Entotheonella*” phosphoenolpyruvate carboxylase (PEPC) sequences, with an *e*-value cutoff of 10^-5^, against the non-redundant protein database of NCBI (Altschul et al., 1990). For phylogenetic analysis, a total of 277 reviewed PEPC sequences were retrieved from the Uniprot database (Consortium, 2015). A second round of BLASTP analysis was carried out using downloaded PEPC sequences as the local database to discard sequences that were largely distant to “*Entotheonella*,” resulting in a reference dataset of 55 high-quality sequences ranging from bacteria to higher plants. Sequences were then aligned using MUSCLE (Edgar, 2004). The alignment was then manually inspected and corrected, resulting in a final alignment with 364 amino acid sites included. A maximum-likelihood tree was reconstructed based on the JTT+G model with a bootstrap value of 1000 in MEGA6 (Tamura et al., 2013).

### Nucleotide Sequence Accession Numbers

The draft genomes of v4.2 and v4.3 were deposited at the European Nucleotide Archive (ENA) under accession PRJEB12598 and PRJEB12599, respectively. Additionally, the annotated genomes are available from the RAST guest account^2^ (username guest; password guest) with accession numbers 93171.12 for v4.2 and 93171.13 for v4.3.

## Results And Discussion

### Genome Recovery

Metagenome bins generated by MetaBAT with a minimum size of 0.5 Mb were submitted to JSpeciesWS Online to target taxa of interest. Two metagenome bins, v4.2 and v4.3, were found to be closely related to the two known “*Entotheonella*” phylotypes/species, respectively (Wilson et al., 2014). Based on the correlation Z-score to all published whole and draft genomes, bin v4.2 and “*Candidatus Entotheonella gemina*” TSY2 (hereafter TSY2) had a Z-Score of 0.995, while the Z-score between bin v4.3 and “*Candidatus Entotheonella factor*” TSY1 (hereafter TSY1) reached 0.996. A Z-score >0.989 suggested two strains were closely related. The ANI between v4.2 and TSY2 was 99.90%. Meanwhile, the ANI between v4.3 and TSY1 was 99.91%. ANI higher than 96% strongly indicates two genomes represent the same species (Richter and Rosselló-Móra, 2009).

General information for binned genomes and their close relatives is summarized in **Table 1**. Compared with TSY1, the completeness of v4.3 was lower, whereas v4.2 had greater completeness than TSY2. The contamination (multiple copies of essential genes) rates of v4.2 and v4.3 were both less than 4%. Overall, the quality of the two metagenome bins in this study met the requirement for reliable genome annotation and comparison with TSY1 and TSY2. Due to the high genome sequence similarities between genomes from the Japan Sea and the South China Sea, the functional annotation profiles of the genomes from the two geographical locations were reasonably coherent. Also, because of the considerable number of hypothetical proteins, the annotation profiles of the two “*Entotheonella*” phylotypes were largely similar. Hence, the subsequent analysis focused on the shared features of all four genomes, which comprised 62.4% of the total unique features (3182 out of 5099 non-hypothetical proteins). In this study, we re-annotated TSY1 and TSY2, which were previously annotated by a RefSeq pipeline (Wilson et al., 2014). In contrast to the RefSeq pipeline, the RASTtk pipeline consisted of steps of re-annotating hypothetical proteins and an algorithm for alternate gene calling. Therefore, the proportion of hypothetical proteins derived from the RASTtk pipeline was less than that from the RefSeq pipeline. The functions that could not be annotated by the RefSeq pipeline but were annotated by the RASTtk pipeline are listed in Supplementary Data Sheet 1.

**Table 1:** General information for metagenome bins obtained in this study and their close relatives.

Taxon^a^	v4.3	TSY1	v4.2	TSY2
Genome size (Mb)	6.1	8.9	7.9	8.5
G+C content (%)	56.48	55.79	56.26	55.55
N50 (kb)	5.1	8.4	6.9	4.2
No. of contigs	1354	1596	1378	2592
No. of CDSs^b^	6618	8397 (8438)	8752	8748 (8989)
Completeness (%)	73.6	93.4	82.1	77.3
No. of hypothetical proteins^b^	4063	4790 (7096)	5772	5359 (7935)
No. of seed subsystems	766	828	813	839

*^a^v4.3, v4.2 and were obtained in this study. TSY1 (AZHW01000000) and TSY2 (AZHX01000000) were from the Japan Sea sponge Theonella swinhoei. ^b^Genomes of TSY1 and TSY2 were re-annotated in this study using a RASTtk pipeline. The counts of CDSs and hypothetical proteins derived from a RefSeq pipeline are shown in parentheses.*

According to the output of EDGAR, the pan genome of “*Entotheonella*” contained 18,825 genes, 13,942 of which were annotated as hypothetical proteins. The core gene set contained 2946 genes, 1672 of which were annotated as hypothetical proteins (Supplementary Data Sheet 2). Besides the core gene set, we identified 1648, 1889, 678, and 459 singletons from TSY1, TSY2, v4.2, and v4.3, respectively. However, only 447 (9.6%) of the singletons were not hypothetical proteins. Annotations of these 447 genes are provided in Supplementary Data Sheet 3.

### Mixotrophy of “*Entotheonella*”

**FIGURE 1:**
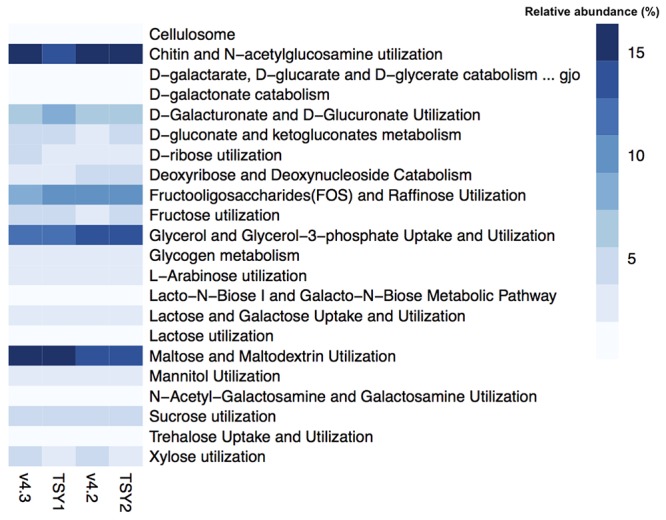
**Relative abundance of features related to the utilization of various carbon sources**.

In general, “*Entotheonella*” contains the genomic capacity for an aerobic heterotrophic lifestyle. Near-complete pathways for glycolysis, the tricarboxylic acid cycle (TCA), the pentose phosphate pathway, and oxidative phosphorylation could be identified from the four “*Entotheonella*” genomes, including v4.2 and v4.3 from the South China Sea, and TSY1 and TSY2 from the Japan Sea (Supplementary Figure S1). The SEED subsystem used the general term “feature” to describe any genomic region with some annotated functions. According to the feature counts, about 10% of “*Entotheonella*” features were related to the utilization of various carbon sources. For carbon source utilization, the most abundant subsystem was “Chitin and N-acetylglucosamine utilization” (**Figure 1**). The highly efficient uptake of dissolved and particulate organic matter by sponges could provide the microbial communities with various forms of carbon source (De Goeij et al., 2013). The ability to utilize a wide range of carbon sources could be beneficial for “*Entotheonella.*” The capacity to degrade recalcitrant carbon sources (e.g., chitin and cellulose) would also benefit the host and other symbiotic members by increasing the availability of more labile carbon sources. Similarly, the ability to degrade N-acetylglucosamine was found in another iconic phylum of sponge symbionts, “*Poribacteria*” (Siegl et al., 2011). The source of chitin and cellulose in the sponge mesohyl may be food particles, which are derived from particulate organic matter taken up by pinacocytes and choanocytes of sponges (Maldonado et al., 2012).

Urea and other nitrogen-containing metabolic products are considered to be important nitrogen sources of sponge symbionts (Webster and Thomas, 2016). Urease (EC 3.5.1.5), urease accessory proteins, urea channels, and urea transporters were found in all “*Entotheonella*” genomes. We also investigated the proteinogenic amino acid biosynthesis potential; however, we only found features related to the biosynthesis of Cys, Met, Lys, Thr and Ser, implying “*Entotheonella*” relies on exogenous amino acid supply, which could be available from its sponge host or the symbiotic microbiota. Accordingly, we found 11 peptidases that were present in all “*Entotheonella*” genomes. These peptidases belonged to aminopeptidases (EC 3.4.11.-), metallocarboxypeptidases (EC 3.4.17.-), omega peptidases (EC 3.4.19.-), serine endopeptidase (EC 3.4.21.-), and signal peptidase. Features related to peptide transportation, such as ABC transporters of oligopeptides (TC 3.A.1.5.1), dipeptides (TC 3.A.1.5.2), and branched-chain amino acids (TC 3.A.1.4.1) were also found in all “*Entotheonella*” genomes.

**FIGURE 2:**
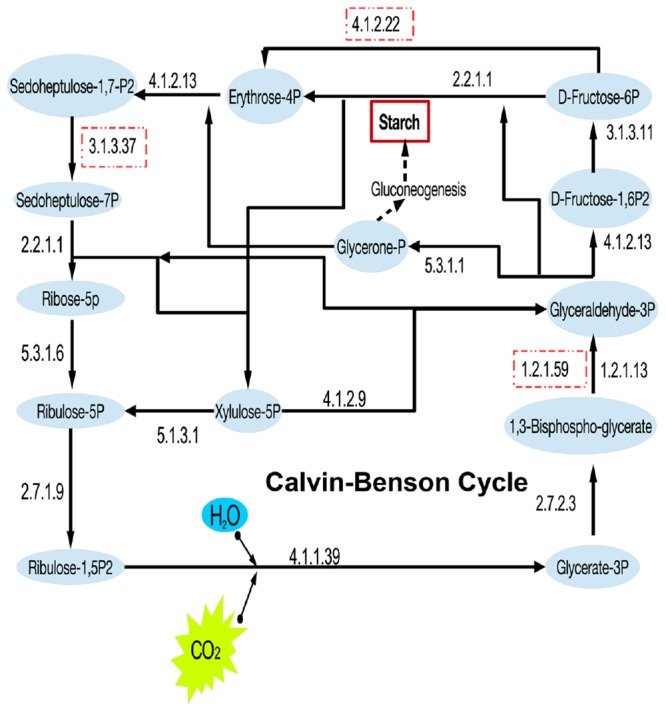
**Reconstruction of the Calvin–Benson cycle in “*Entotheonella*”.** The EC numbers of key enzymes are shown. The dashed squares indicate the enzyme was missing in at least one genome.

Besides the heterotrophic traits, the existence of RuBisCO indicated the carbon fixation potential of “*Entotheonella.*” A near-complete Calvin–Benson cycle was reconstructed in this study (**Figure 2**). Some missing genes could be due to incompleteness of the current genomes. Carbonic anhydrase (EC 4.2.1.1) was also found in all “*Entotheonella*” genomes. This enzyme aids in concentrating CO_2_ by catalyzing CO_2_ hydration, a simple but physiologically relevant reaction in all kingdoms of life (Capasso and Supuran, 2015).

**FIGURE 3:**
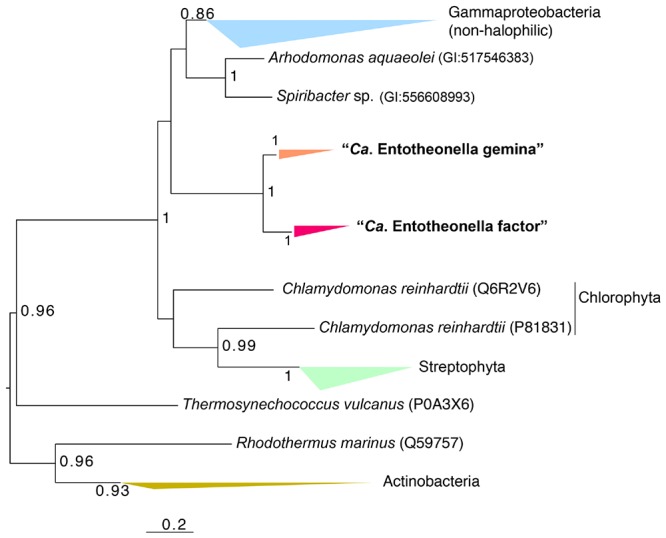
**Maximum-likelihood phylogenetic tree of phosphoenolpyruvate carboxylase (PEPC) genes of “*Entotheonella*”.** Bootstrap values over 0.5 are shown. For sequences retrieved from the Uniprot database, the Uniprot ID is shown in parentheses.

Interestingly, a set of genes related to crassulacean acid metabolism (CAM) and the C_4_-dicarboxylic acid cycle existed in all “*Entotheonella*” genomes. It is known that CAM and the C_4_-dicarboxylic acid cycle greatly enhance CO_2_ concentration in higher plants. Nonetheless, particular tissue structures and complex regulation mechanisms are involved in CAM and the C_4_-dicarboxylic acid cycle of plants (Dodd et al., 2002; Langdale, 2011). For “*Entotheonella*,” a possible scenario is that these enzymes coexist for unknown reasons and function in other metabolic pathways, as none of the enzymes is unique to plants. By searching KEGG reference genomes, we found that genes related to CAM existed in the genome of the legume symbiont *Methylobacterium nodulans* as well (Jourand et al., 2004). The key enzyme of CAM and the C_4_-dicarboxylic acid cycle is PEPC (Christin et al., 2014). BLAST analysis showed the PEPC sequences of “*Entotheonella*” were most similar to those of the halophilic Gammaproteobacteria (*Arhodomonas* and “*Spiribacter*”; Adkins et al., 1993; López-Pérez et al., 2013). Phylogenetic analysis showed PEPC of “*Entotheonella*” differed from the homologs of “*Spiribacter*” and *Arhodomonas*, although they shared high sequence similarity (**Figure 3**). The placement of “*Entotheonella*” PEPC was divergent from the Gammaproteobacteria and Viridiplantae, implying the unique evolutionary traits of “*Entotheonella.*”

With a predicted genome size over 9 Mb, “*Entotheonella*” belongs to the bacteria with large genomes (Wilson et al., 2014; and the data^3^). The mechanisms that support the large genomes of “*Entotheonella*” are worth mining. DNA replication is typically demanding with respect to nutrients and energy. For microbes inhabiting niches with scarce nutrients, genome streamlining reduces the minimum requirement for reproduction and thus promotes survival (Giovannoni et al., 2014). Experiments have proven marine sponges can effectively uptake dissolved and particulate organic matter in seawater (Yahel et al., 2003; De Goeij et al., 2008, 2013; Hadas et al., 2009). The organic matter might subsequently serve as communal food particles for sponge cells and microbes. We speculated that “*Entotheonella*” could benefit from communal food particles. Meanwhile, the potential for CO_2_ fixation offers an alternative strategy when carbon sources are in short supply, e.g., competitive carbon uptake from other symbionts. More experiments are needed to determine how important CO_2_ fixation is to “*Entotheonella*” and the whole symbiotic community of *T. swinhoei*.

Low DNA G+C content (<30%) is considered an adaptive trait of planktonic bacteria when facing limited nitrogen sources (Luo and Moran, 2015). With G+C content over 55%, the replication of “*Entotheonella*” DNA is not only energy demanding but also nitrogen demanding. Marine sponges have been shown to serve as a net nitrogen source through remineralization of particulate organic matter (Maldonado et al., 2012). Hence, “*Entotheonella*” in sponge mesohyl may face less nitrogen limitation than those low G+C content planktonic bacteria. In addition to the inorganic nitrogen sources, the symbiotic community may also provide organic nitrogen sources for “*Entotheonella.*” The organic nitrogen sources in sponge mesohyl could be the leaky products from the metabolism of other symbionts (Giovannoni et al., 2014) and the sponge cell detritus (De Goeij et al., 2013).

### Adaption of “*Entotheonella*” to Anaerobic Conditions in the Sponge Body

**FIGURE 4:**
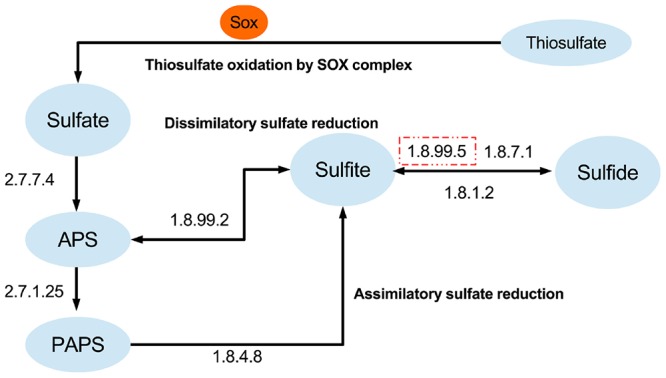
**Sulfate-reducing and sulfur-oxidizing metabolism capacity of “*Entotheonella*”.** The dashed square indicates the enzyme was missing in at least one genome.

Apart from carbon fixation potential, “*Entotheonella*” genomes encode enzymes for sulfate reduction. A near complete dissimilatory sulfate reduction pathway was reconstructed from all “*Entotheonella*” genomes (**Figure 4**). The dissimilatory sulfate reduction potential may supply the energy that is needed in the Calvin–Benson cycle. Compared with the newly found sulfur-oxidizing bacterium in deep-sea glass sponge (Tian et al., 2016), TSY1 and v4.3 lacked dissimilatory sulfite reductase (EC 1.8.99.5), which might be due to the incompleteness of the genomes. In addition, the Sox complex and a complete assimilatory sulfate reduction pathway were found in all four genomes. When “*Entotheonella*” was recognized as a genus of Deltaproteobacteria, a mixed culture of “*Entotheonella*” and other bacteria could be acquired using media designed for sulfate-reducing bacteria (Schmidt et al., 2000). Our finding of sulfate-reduction potential in “*Entotheonella*” echoed this research. Anaerobic zones existed in actively pumping sponges, and activities of both sulfate reduction and anaerobic ammonia oxidation were detected (Hoffmann et al., 2005; Mohamed et al., 2010). The chemotrophic ability of “*Entotheonella*” ensures its energy supply when oxygen is scarce. Additionally, carboxylic acids derived from the sulfate reduction process might be transferred to the host (Hoffmann et al., 2005).

Features related to anaerobic respiratory reductases were detected in “*Entotheonella.*” The features shared by all “*Entotheonella*” genomes included flavodoxin reductases, anaerobic dehydrogenases, and ferredoxin reductase. Interestingly, “*Entotheonella*” possessed Nar, Nir, Nor, and Nos gene clusters, which implied it could complete anaerobic respiration through denitrification. Additionally, NnrA was found only in “*Candidatus Entotheonella factor*,” i.e., TSY1 and v4.3.

Adaptive traits of sponge symbionts to anaerobic conditions are not confined to “*Entotheonella.*” A recent study showed that a combination of aerobic and microaerophilic states could increase the diversity and novelty of cultivable microbes from *T. swinhoei*, which suggested that the microbes in *T. swinhoei* faced variable oxygen levels (Lavy et al., 2014). Study of six sponge metagenomes has highlighted the enrichment of adaptive traits to anaerobic conditions, such as nitrate respiration (Fan et al., 2012). In “*Poribacteria*,” genomic traits related to a lifestyle under anaerobic conditions, including nitrite reductase (EC 1.7.2.1), nitric oxide reductase (EC 1.7.99.1), and the Wood–Ljungdahl pathway (anoxic CO_2_ fixation) have been found (Siegl et al., 2011).

### Resilience of “*Entotheonella*”

**FIGURE 5:**
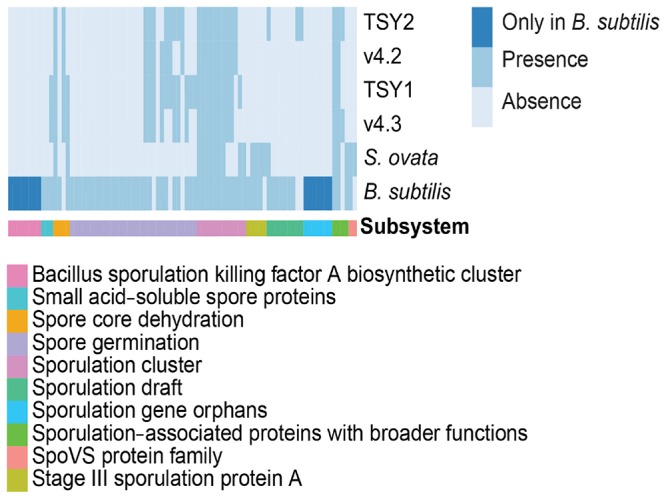
**Comparison of sporulation features across *B. subtilis* (GCA_000009045.1), *Sporomusa ovata* (ASXP01000000), and four “*Entotheonella*” genomes.** *B. subtilis* and *Sporomusa ovata* were re-annotated using a RASTtk pipeline.

Features of endospore formation were found in “*Entotheonella*” genomes. Until now, the only known Gram-negative bacterium that could form endospores was *Sporomusa ovata* (Poehlein et al., 2013). In comparison with *S. ovata* and the model species *Bacillus subtilis*, “*Entotheonella*” genomes possessed the coding potential for spore core dehydration, spore germination, and sporulation clusters (**Figure 5**). Bacterial endospores are the most durable cells in nature (Nicholson et al., 2000). The potential for endospore formation could strengthen the resistance of “*Entotheonella*” against environmental stress and increase its ability to thrive in a diverse range of niches. One possible scenario is that *T. swinhoei* harbors dense microbiota, and hence the intra-/inter-species competition is fiercer than that in planktonic communities. By forming endospores, some “*Entotheonella*” cells could survive and relocate to other hosts.

The “*Entotheonella*” 16S rRNA gene could be detected in sponges and seawater from different geographical locations (Wilson et al., 2014). However, microscopic evidence for the presence of “*Entotheonella*” has been found only in lithistid sponges (e.g., *Theonella* and *Discodermia*; Schmidt et al., 2000; Brück et al., 2008; Wilson et al., 2014). For instance, *Aplysina aerophoba* yielded PCR products of the “*Entotheonella*” 16S rRNA gene but no filamentous bacteria were observed during transmission electron microscopy (Friedrich et al., 2001). Based on the sporulation-related features of “*Entotheonella*,” we hypothesized that its spores are widespread and the unknown cue for spore germination lies in Lithistida sponges. The genetic elements encoding the endospore-forming process are flexible in composition, conserved in evolution, and sophisticated in regulation. Even in the well-studied *B. subtilis*, more information is needed to fully understand the endospore-forming process (Hutchison et al., 2014). Sporulation-related features in the “*Entotheonella*” genomes may only represent the tip of its development course. It would be interesting to monitor the development of *T. swinhoei* and investigate when its filamentous partner appears.

“*Entotheonella*” possessed abundant genomic features of metal resistance, including resistance to As, Co, Cu, Hg, and Zn. Beta-lactamase and multi-drug resistance eﬄux pumps were the main mechanisms of antibiotic resistance. A recent attempt at culturing *T. swinhoei* symbiotic bacteria yielded 12 operational taxonomic units that were tolerant to high arsenic concentrations (Keren et al., 2015). The resistance to As and other heavy metals found within ‘*Entotheonella*’ might be beneficial not only for these organisms but also for the *T. swinhoei* symbiotic community, as sponges and their symbionts are frequently exposed to toxic matter in seawater (De Goeij et al., 2013).

### Interaction between *“Entotheonella*” and Other Organisms

**FIGURE 6:**
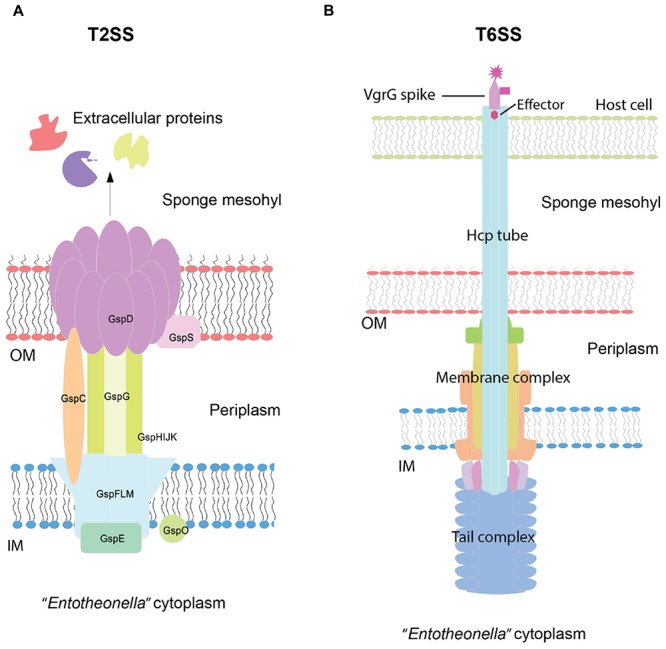
**Schematic view of the structures and mechanisms of Type II (T2SS) (A) and Type VI secretion systems (T6SS) (B).** The structural assembly was predicted based on the gene content of “*Entotheonella*” draft genomes and visualized based on the KEGG diagram and the review of Costa et al. (2015). OM, outer membrane; IM, inner membrane. According to the review of Costa et al. (2015), in T2SSs, the GspC protein recruits folded exoproteins from the periplasm to secretin (GspD). GspE acts as ATPase and facilitates the assembly of the periplasmic pseudopilus (GspG) through the IM. The exoproteins are pushed by the pseudopilus through the secretin channel. In T6SSs, effectors are recruited by VgrG and pass through the haemolysin co-regulated protein (Hcp) tube. The sheath contraction is triggered by an unknown extracellular signal, resulting in the ejection of the effectors into targeting cells.

Eukaryotic-like proteins (ELPs) are well known as important factors involved in sponge–microbe interactions (Fan et al., 2012; Nguyen et al., 2014). In “*Entotheonella*” genomes, ELPs such as ankyrin repeat proteins (ARP), leucine-rich repeats (LRR), and tetratricopeptide repeats (TPR) were found (Supplementary Table S1). Other than ELPs, secretion systems of Gram-negative bacteria play key roles in response to environmental factors and interaction with other macro-/micro-organisms (Costa et al., 2015). Type II secretion systems (T2SSs) and Type VI secretion systems (T6SSs) represented the major protein secretion systems unveiled in this study (Supplementary Table S2; **Figure 6**).

In terms of T2SSs, we found general secretion pathway proteins (Gsps) and features related to the Widespread Colonization Island. T2SSs are found in both clinical pathogens and non-pathogens and usually require 12–15 Gsps to form the double-membrane-spanning structure (Costa et al., 2015). In our study, we found 14 Gsps from v4.2, v4.3, and TSY1. For unknown reasons, TSY2 was devoid of Gsps. T2SSs secrete folded proteins from the periplasm into the extracellular environment (**Figure 6A**). Extracellular proteins/enzymes are important for microbes to utilize the organic matter in a certain niche or interact with other organisms. Some extracellular proteolytic enzymes, mainly of the metalloprotease group, have been found to be virulence factors (Costa et al., 2015). Only one secreted collagenase (EC 3.4.24.3) was found in “*Entotheonella*,” whereas the known sponge pathogen *Pseudoalteromonas agarivorans* NW4327 contained 30 secreted serine proteases and metalloproteases (Choudhury et al., 2015). Here we hypothesize that the ability to degrade collagen might be essential for “*Entotheonella*” to obtain space in the sponge mesohyl.

The Widespread Colonization Island represents an ancient and a new subtype of Type II secretion, which encompasses the *tad* (tight adherence) locus for colonization of surfaces and biofilm formation (Planet et al., 2003). The Widespread Colonization Island also represents a hotspot of horizontal gene transfer, which might function in the evolution and diversification of “*Entotheonella.*” In a study of *Actinobacillus*, at least 12 of the *tad* genes were necessary to generate the adherence-related phenotypes (Tomich et al., 2007). In this study, we identified features related to 17 *tad* genes, suggesting “*Entotheonella*” could form tight adherence, which might contribute to its stable residence in sponges or other types of host–microbe interactions. Nevertheless, the “*Entotheonella*” cells were free of fibrils and flagella (Wilson et al., 2014). The regulation and structure of the “*Entotheonella*”-derived *tad* locus need further investigation.

T6SSs were first discovered in *Vibrio cholerae* (Pukatzki et al., 2006). The needle-like structure could inject effectors and toxic proteins into other cells (mostly host/eukaryotic cells) and then kill the cells (**Figure 6B**). All the core components of T6SSs (Boyer et al., 2009) were detected in “*Candidatus Entotheonella gemina*” (TSY2 and v4.2), whereas the spike protein VgrG was missing in “*Candidatus Entotheonella factor*” (TSY1 and v4.3). A previous study of the sponge pathogen *P. agarivorans* NW4327 revealed the existence of 19 genes related to T6SSs (Choudhury et al., 2015). This study is the second report of T6SSs in a sponge symbiont. Nevertheless, T6SSs are widespread in nature and not confined to known pathogens (Costa et al., 2015). In some cases, T6SSs can kill prokaryotic cells as well (Miyata et al., 2013). A recent pyrosequencing study of the protistan community in *T. swinhoei* revealed the existence of radiolarians, which might prey on symbiotic prokaryotes (He et al., 2014). We hypothesized that a potential benefit for “*Entotheonella*” of having T6SSs might be the ability to fight against zooplankton predators. Currently, there is no report of *T. swinhoei* disease and no “*Entotheonella*” features have been found under SEED subcategory “Toxins and superantigens.” Its uncultured status largely restricts the biochemical and physiological study of “*Entotheonella.*” How virulent “*Entotheonella*” could be to its hosts and how T6SSs are regulated in “*Entotheonella”* remain unknown. The present evidence does not support that T6SSs are related to the virulence of “*Entotheonella.*” Nonetheless, it is likely that T6SSs make “*Entotheonella*” a competitive member of the symbiotic community.

## Summary

The uncultured status of “*Entotheonella*” means it remains cryptic in terms of life history, physiology, ecological roles, and association with its sponge host. The four draft genomes, despite being fragmentary and not thoroughly annotated, offered new insights into “*Entotheonella*” in *T. swinhoei*, e.g., mixotrophic traits (utilization of various carbon sources and CO_2_ fixation), adaptation to the anaerobic environment, resistance to threats, and interaction with other organisms (**Table 2**).

**Table 2:** Summary of functional traits of “*Entotheonella*”.

Functional traits	Interpretation
Calvin–Benson cycle	CO_2_ fixation
Utilization of various carbon sources	Adaptation to the diverse organic matter in sponge mesohyl
Anaerobic respiration	Energy supply in anoxic environment
Denitrification	Energy supply in anoxic environment
Sulfate reduction	Energy supply in anoxic environment
Peptidase	Compensation for the reduced amino acid synthesis capacity
Endospore formation	Resistance to environmental threats; widespread distribution
Metal resistance	Resistance to environmental threats
Antibiotic resistance	Competition in symbiotic communities; resistance to environmental threats
Eukaryotic-like proteins	Host–microbe recognition
Type II secretion system	Interaction with host environment
Widespread Colonization Island	Tight adhesion
Type VI secretion system	Host–microbe interaction, microbe–microbe competition

Despite the differences between the South China Sea and the Japan Sea in terms of temperature, salinity, and primary productivity, the “*Entotheonella*” strains from the two sea areas showed high intraspecies genomic coherence. Furthermore, future studies addressing the relationship between “*Entotheonella*” genetic diversity and host morphological features are essential for understanding the diversification of “*Entotheonella.*” The interior of *T. swinhoei* investigated by Wilson et al. (2014) and us was yellow. *T. swinhoei* with a white interior was reported to harbor a different phylotype, “*Candidatus Entotheonella serta*” (genome not available; Ueoka et al., 2015). Comparative studies on white and yellow *T. swinhoei* genomes and their “*Entotheonella*” genomes will yield more valuable insights into symbiont diversification and sponge–symbiont interactions.

The genome information also provided clues for improving the strategy of cultivating “*Entotheonella*” in the lab. Firstly, genome analysis of “*Entotheonella*” suggests it does not favor one specific carbon source but can utilize a wide range of carbohydrates. The combination of various carbon sources might be important for its metabolism and growth. Secondly, although “*Entotheonella*” has a large and distinct secondary metabolism repertoire, it is able to synthesize only five proteinogenic amino acids (Cys, Met, Lys, Thr and Ser). Thus, providing free amino acids would be crucial for cultivating “*Entotheonella*” due to its deficiency in synthesizing all proteinogenic amino acids. Thirdly, the anaerobic respiration reductases and sulfate-reduction capacity suggested “*Entotheonella*” faced variable oxygen levels in *T. swinhoei*. The shift between anaerobic and aerobic conditions might be important for the activity and metabolic regulation of “*Entotheonella.*”

## Author Contributions

FL and ZL designed the study. FL undertook metagenome binning and genome analysis. JL finished the metagenome sequencing and assembling. GF sampled the sponges and extracted the DNA. All authors discussed and wrote the manuscript together.

## Conflict of Interest Statement

The authors declare that the research was conducted in the absence of any commercial or financial relationships that could be construed as a potential conflict of interest.

## Footnotes

1 https://github.com/lmrodriguezr/enveomics-gui

2 http://rast.nmpdr.org/

3 http://www.genomesize.com/prokaryotes
