# Supplementary material for: New Genomic Insights into “Entotheonella” Symbionts in Theonella swinhoei: Mixotrophy, Anaerobic Adaptation, Resilience, and Interaction
Source: Front Microbiol. 2016 Aug 25;7:1333. doi: 10.3389/fmicb.2016.01333 (PMC4996862; doi:10.3389/fmicb.2016.01333)
Supplement: Supplementary file 1 [file Presentation_1.PDF]

Supplementary Information

New genomic insights into “*Entotheonella*” symbionts in *Theonella swinhoei*: mixotrophy, anaerobic adaptation, resilience and interaction

Fang Liu, Jinlong Li, Guofang Feng, Zhiyong Li

Marine Biotechnology Laboratory, State Key Laboratory of Microbial Metabolism and School of Life Sciences and Biotechnology, Shanghai Jiao Tong University, Shanghai 200240, P.R. China

* Correspondence
Zhiyong Li:
Marine Biotechnology Laboratory, School of Life Sciences and Biotechnology, Shanghai Jiao Tong University, 800 Dongchuan Road, Shanghai 200240, People’s Republic of China
E-mail: zyli@sjtu.edu.cn; Tel.: 86-21-34204036; Fax: 86-21-34205709

Supplementary scripts

Metagenome binning using MetaBAT:

Step 1: run MetaBAT with default settings (sensitive mode)

```
runMetaBat.sh metagenome-assembly.fa metagenome-assembly.sorted.bam
```

Step 2: run MetaBAT with specific mode in the same folder (the *.depth.txt, *.TNF, and *.distance were generated in step 1)

```
metabat -i metagenome-assembly.fa -a metagenome-assembly.fa.depth.txt -o output-folder --
specific -l -v -m 2000 --saveTNF saved-metagenome-assembly.fa.depth.txt.TNF  --
saveDistance saved-metagenome-assembly.fa.depth.txt.distance
```

ani.rb. and hmm.essential.rb were executed in enveomics-GUI which didn't require any command lines. The missing essential genes indicated by hmm.essential.rb were re-checked in the RASTtk annotation of four draft genomes.

Genome annotation based on RASTtk

#Double click RASTtk.app and navigate to terminal window. Here we use v4.2 as an example to show the command lines
#One could perform the annotation using RASTtk pipeline on RAST website as well

```
rast-create-genome --scientific-name "Entotheonella sp." --genetic-code 11 --domain Bacteria
--contigs v4.2.fa > 4.2.gto
rast-call-features-rRNA-SEED < 4.2.gto > GTO.2
rast-call-features-tRNA-trnascan < GTO.2 > GTO.3
rast-call-features-repeat-region-SEED < GTO.3 > GTO.4
rast-call-features-selenoprotein < GTO.4 > GTO.5
rast-call-features-pyrrolysoprotein < GTO.5 > GTO.6
rast-call-features-crispr < GTO.6 > GTO.7
rast-call-features-CDS-prodigal < GTO.7 > GTO.8
rast-call-features-CDS-glimmer3 < GTO.8 > GTO.9
rast-call-features-ProtoCDS-kmer-v2 < GTO.9 > GTO.10
rast-call-features-ProtoCDS-kmer-v1 < GTO.10 > GTO.11
rast-annotate-proteins-kmer-v2 < GTO.11 > GTO.12
rast-annotate-proteins-kmer-v1 -H < GTO.12 > GTO.13
rast-annotate-proteins-similarity -H < GTO.13 > GTO.14
rast-resolve-overlapping-features < GTO.14 > GTO.15
rast-export-genome feature_data < GTO.15 > 4.2.table
```

Figure S1. KEGG mapping results of glycolysis (brown), TCA cycle (green), the pentose phosphate pathway (orange), and the oxidative phosphorylation (red) in “*Entotheonella*” genomes.

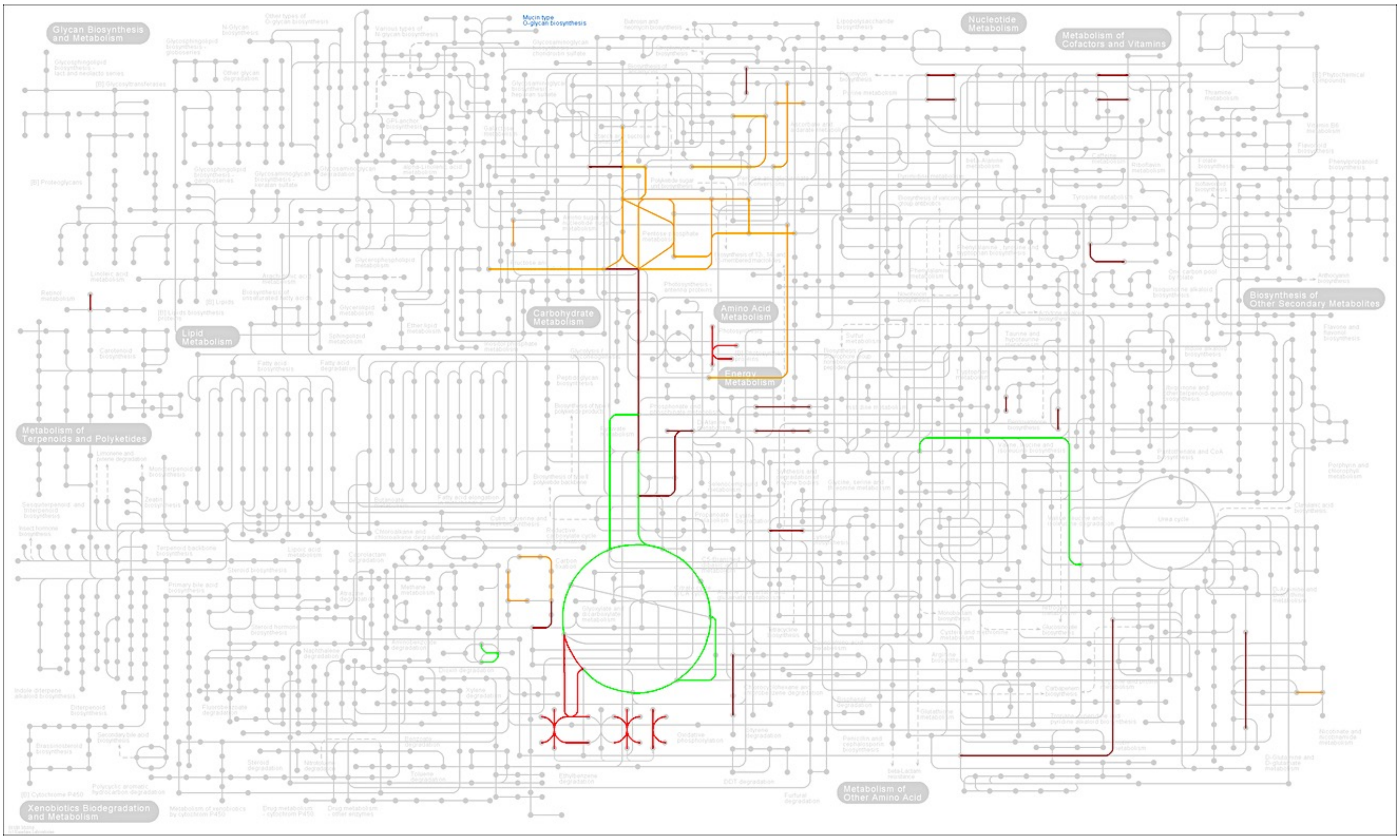

Table S1. Representative Eukaryotic-like proteins in "*Entotheonella*"

	Uniprot ID [a]	Protein	v4.2	TSY2	v4.3	TSY1
TPR	D5UPJ5	1D-myo-inositol 2-amino-2-deoxy-alpha-D-glucopyranoside ligase	278.5	235.3	282.3	282.3
LRR	W4LJP9	Predicted GTP-binding protein			1282.3	1542.3
	W4MAU1	Predicted GTP-binding protein	1033.9	1021.9		
ARP	M9WXW8	Ankyrin repeat domain protein	50.8			
	A0A0C2QBL3	Ankyrin		72.4	49.7	49.7

a. Eukaryotic-like protein sequences were downloaded from Uniprot database. BLASTP program was used to search the Eukaryotic-like proteins in query genomes (e-value < 10^{-5}). The best blast hit and the bitscore of each query genome was showed.

Table S2. Distribution of genes related to secretion systems in "*Entotheonella*"

		v4.3	TSY1	v4.2	TSY2
Gsp	GspD	+	+	+	-
	GspE	+	+	+	-
	GspA	+	+	+	-
	GspK	+	+	+	-
	GspL	+	+	+	-
	GspN	+	+	+	-
	GspF	+	+	+	-
	GspC	+	+	+	-
	GspG	+	+	+	-
	GspJ	+	+	+	-
	GspI	+	+	+	-
	GspB	+	+	+	-
	GspM	+	+	+	-
	GspH	+	+	+	-
WCI	TadA	+	+	+	+
	TadC	+	+	+	+
	rcpA	+	+	+	+
	TadB	+	+	+	+
	rcpC	+	+	+	+
	Flp pilus assembly protein	+	+	+	+
	TadZ	+	+	+	+
	ATPase with chaperone activity	+	+	+	+
	TadZ/CpaE	+	+	+	+
	TadD	+	+	+	+
	CpaD	+	+	+	+
	Von Willebrand factor type A domain protein	+	+	+	+
	TadG	+	+	+	+
	ATP-dependent helicase	+	+	+	+
	TadV	-	+	+	+
	TadE	-	-	+	+
	TadF	-	-	+	+
T6SS	*ClpB protein	+	+	+	+
	*ImpH/VasB	+	+	+	+
	*ImpA	+	+	+	+
	*ImpJ/VasE	+	+	+	+
	*IcmF-related protein	+	+	+	+
	*ImpG/VasA	+	+	+	+
	*ImpC	+	+	+	+
	*ImpB	+	+	+	+
	*ImpI/VasC	+	+	+	+

*ImpF	+	+	+	+
* lipoprotein/VasD	+	+	+	+
*dotU	+	+	+	+
*VgrG protein	-	-	+	+
*ImpD	+	+	+	+
*Secreted protein Hcp	+	+	+	+
Sigma-54 dependent transcriptional regulator	+	+	+	+
Protein phosphatase ImpM	+	+	+	-
ImpN	+	+	-	-
VasI	-	-	+	+
ImpE	-	-	+	+

* Core elements of T6SS
